# Active metabolites of dipyrone induce a redox-dependent activation of the ion channels TRPA1 and TRPV1

**DOI:** 10.1097/PR9.0000000000000720

**Published:** 2019-04-09

**Authors:** Stefan Alexander Schenk, Felicia Dick, Christine Herzog, Mirjam Jeanette Eberhardt, Andreas Leffler

**Affiliations:** Department of Anesthesiology and Intensive Care Medicine, Hannover Medical School, Hannover, Germany

**Keywords:** Dipyrone, Metamizol, Analgesia, TRPV1, TRPA1

## Abstract

**Introduction:**

The nonopioid analgesic and antipyretic dipyrone (metamizol) is frequently used worldwide. Dipyrone is a prodrug, and the metabolites 4-N-methylaminoantipyrine (MAA) and 4-aminoantipyrine (AA) seem to induce analgesia and antipyresia in part by inhibiting cyclooxygenase. In mice, however, the analgesic effect of dipyrone also seems to depend on the ion channel TRPA1. In this study, we explored the effects of dipyrone and its active metabolites on recombinant and native TRPA1 and TRPV1 channels.

**Methods:**

Constructs human (h) TRPA1 and TRPV1 were expressed in HEK293 cells, and dorsal root ganglion neurons were isolated from adult mice. Effects of dipyrone, MAA, and AA were explored by means of whole-cell patch clamp recordings and ratiometric calcium imaging.

**Results:**

Dipyrone failed to activate both hTRPA1 and hTRPV1. However, both MAA and AA evoked small outwardly rectifying membrane currents and an increase of intracellular calcium in cells expressing hTRPA1 or hTRPV1. MAA also sensitized both channels and thus potentiated inward currents induced by carvacrol (hTRPA1) and protons (hTRPV1). MAA-induced activation was inhibited by the antioxidant 10-mM glutathione included in the pipette, and the mutant constructs hTRPA1-C621/C641/C665S and hTRPV1-C158A/C391S/C767S were insensitive to both MAA and AA. Mouse dorsal root ganglion neurons exhibited a marginal calcium influx when challenged with MAA.

**Conclusion:**

The metabolites MAA and AA, but not dipyrone itself, activate and sensitize the nociceptive ion channels TRPA1 and TRPV1 in a redox-dependent manner. These effects may be relevant for dipyrone-induced analgesia and antipyresia.

## 1. Introduction

**Figure 1.**
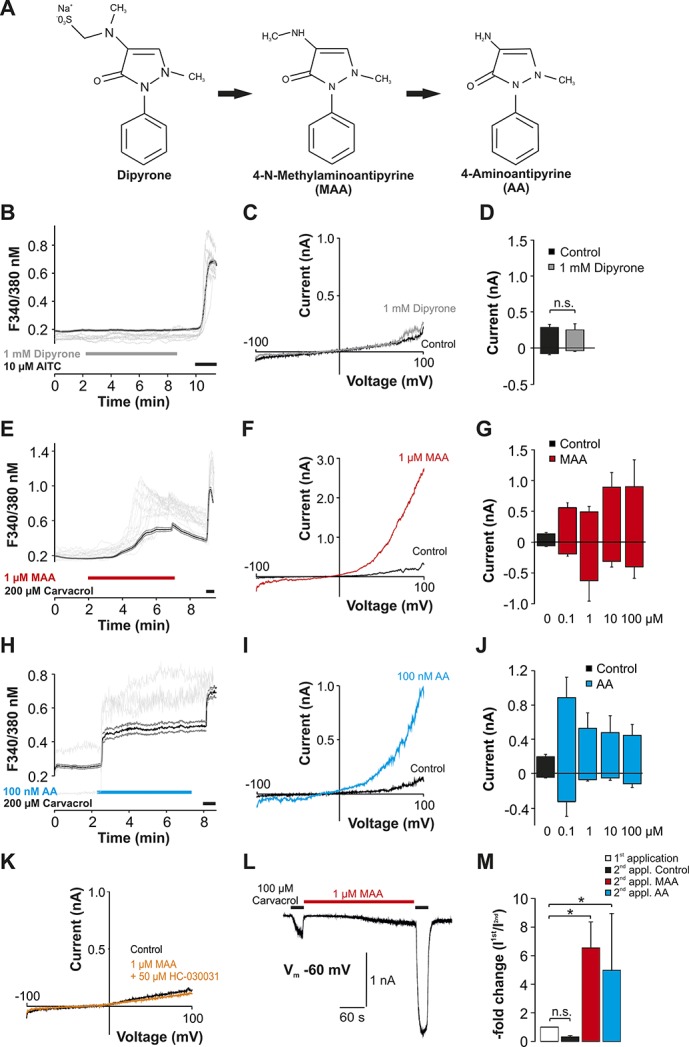
Metabolites of dipyrone activate hTRPA1. (A) Image depicting the metabolism of dipyrone into active metabolites. (B, E, H) Effects of dipyrone (B, 1 mM), MAA (E, 1 µM), and AA (H, 100 nM AA) on intracellular calcium expressed as ratio F340/380 nm in hTRPA1-HEK 293 cells. Dipyrone and metabolites were applied for 5 minutes followed by application of either AITC or carvacrol to confirm expression of hTRPA1. The black lines represent the average (mean ± SEM) of recorded cells, and the gray lines represent typical original traces from individual cells. (C, F, I) Representative patch clamp traces during 500 ms-long voltage ramps ranging from −100 mV to 100 mV in HEK293 cells expressing hTRPA1. The ramps were performed during application of either control solution, 1 mM dipyrone (C), 1 µM MAA (F), or 100 nM AA (I). Expression of hTRPA1 was confirmed by application of carvacrol (traces not shown). (D, G, J) Average (mean ± SEM) current amplitudes of currents at −100 and 100 mV during voltage ramps by different concentrations of dipyrone (D, n.s., not significant; Student paired *t* test), MAA (G), and AA (J). (K) Representative patch clamp trace during 500 ms-long voltage ramps ranging from −100 mV to 100 mV in a HEK 293 cell expressing hTRPA1 during application of control solution or MAA in combination with 50 µM of the TRPA1-antagonist HC-030031. (L) Representative current traces in hTRPA1-HEK293 cells during 2 consecutive applications of carvacrol (100 µM). One micromolar MAA was applied for about 5 minutes between the 2 applications of carvacrol. (M) Average inward currents elicited by the second application of carvacrol, expressed as average “-fold increase” calculated by normalizing the peak currents amplitudes of the second carvacrol-induced current with the first one. n.s., not significant; Student paired *t* test. *P < 0.05.

Dipyrone (metamizol) is an effective analgesic and antipyretic that is rapidly hydrolysed and converted into the metabolites MAA and AA in vivo, both known to inhibit cyclooxygenase (Fig. 1).^5,8,11,13,15^ Dipyrone itself inhibits activation of the irritant receptor TRPA1 by reactive compounds, and dipyrone-induced analgesia is abrogated in mice lacking TRPA1.^12^ TRPA1 is also critical for analgesia and hypothermia induced by acetaminophen, presumably due to an activation of TRPA1 by reactive metabolites.^1,6^ Both MAA and AA are reactive and may thus modify redox-sensitive proteins.^13^ Both TRPA1 and the capsaicin receptor TRPV1 are redox-sensitive, a property which in both ion channels mainly depend on N-terminal cysteines.^2,4,14^ Considering that dipyrone is rapidly hydrolysed to MAA after intake, we hypothesized that the MAA and AA might as well modulate TRPA1 and TRPV1. Therefore, we examined the effects of dipyrone, MAA, and AA on recombinant and native TRPA1 and TRPV1 channels.

## 2. Methods

### 2.1. Chemicals

Dipyrone, reduced glutathione (Sigma-Aldrich, Taufkirchen, Germany), 4-N-methylaminoantipyrine, and 4-aminoantipyrine (Sanofi-Aventis, Frankfurt, Germany). L-dithiothreitol, capsaicin, and carvacrol (Sigma-Aldrich). Allyl isothiocyanate (AITC; Merck, Darmstadt, Germany). BCTC (4-(3-Chloro-2-pyridinyl)-N-[4-(1,1-dimethylethyl)phenyl]-1-piperazinecarboxamide) and HC030031 (Tocris, Bristol, United Kingdom).

### 2.2. Cell culture

Stable cell lines with hTRPA1 or hTRPA1 were cultured and used as described previously.^3^ HEK-293 t cells were cultured in DMEM (D-MEM; Gibco, BRL Life Technologies, Karlsruhe Germany) with 10% FBS (Biochrom, Berlin, Germany) and 100 µg/mL penicillin–streptomycin (Gibco). For transient transfection, cDNAs were transfected with the nanofectin transfection kit (PAA, Pasching, Austria). Cysteine modifications in hTRPA1 and hTRPV1 were introduced by using the QuikChange Lightning Site-Directed Mutagenesis Kit (Agilent Technologies, Waldbronn, Germany). All cysteine exchanges were verified by DNA sequencing (GATC, Konstanz, Germany). Experiments were performed in accordance with the requirements of the local authorities (Hannover, Niedersachsen, Germany).

### 2.3. Electrophysiology

Whole-cell patch clamp was performed using the Patchmaster Software (HEKA Electronics, Lambrecht, Germany) and a HEKA USB 10 amplifier. Pipette solution was prepared using (in mM) NaCl 140, KCl 5, MgCl_2_, CaCl_2_ 1.2, HEPES 10, and glucose 10, pH 7.4. Calcium-free extracellular solution was prepared using (in mM) NaCl 140, KCl 5, MgCl_2_ 2, EGTA 5, HEPES 10, and glucose 10, pH 7.4. Data were sampled at 10 kHz and filtered at 2 kHz. The Fitmaster Software (HEKA Electronics) as well as Origin 7.0273 and Origin 8.5.1 (Origin Lab, Northampton, MA) were used for data analysis. Paired Student *t* test was performed for statistical analyses on dependent variables. *P* < 0.05 was regarded statistically significant.

### 2.4. Ratiometric [Ca^2+^]_i_ measurements

Coverslips were incubated with 4-µM Fura-2-AM and 0.02% pluronic for 45 minutes and then mounted on an inverse microscope (Axio observer D1; Zeiss, Jena, Germany). Cells were superfused with (in mM) NaCl 145, KCl 5, CaCl_2_ 1.25, MgCl_2_ 1, glucose 10, and HEPES 10, pH 7.4. Images were exposed for 20 and 40 ms, respectively, and acquired at 1 Hz with a CCD camera (Cool SNAP EZ; Photometrics, Puchheim, Germany). Data were recorded using VisiView 2.1.1 software (Visitron Systems GmbH, Puchheim, Germany).

### 2.5. Dorsal root ganglion culture

Dorsal root ganglion (DRG) neurons were isolated from adult wild-type C57/BL6 mice. After surgical preparation, ganglia were incubated for 1 hour at 37°C in DMEM (Invitrogen, Darmstadt, Germany) containing 0.6 mg/mL collagenase (type XI) and 3 mg/mL protease (both, Sigma Aldrich, Seelze, Germany) before dissociated neurons were plated onto coverslips coated with poly-d-lysine (0.1 mg/mL for 30 minutes). Cells were cultured (37°C and 5% CO_2_) in serum-free TNB-100 basal medium (Biochrom AG), supplemented with penicillin/streptomycin 100 U/mL.

## 3. Results

### 3.1. MAA and AA, but not dipyrone, activate hTRPA1 and hTRPV1

Dipyrone up to 1 mM did not evoke changes in intracellular calcium (n = 262, Fig. 1B) or increased membrane currents (Fig. 1C, D, n = 7) in HEK293 cells expressing hTRPA1. By contrast, 1 µM of MAA induced a calcium influx (Fig. 1E, n = 92) as well as outwardly rectifying membrane currents monitored during voltage ramps ranging from −100 to +100 mV within 500 ms (Fig. 1F, n = 5). This effect did not obey a clear concentration dependency (Fig. 1G, n = 5–7 for each concentration). AA also induced an increase of intracellular calcium (Fig. 1H, n = 109) and membrane currents (Fig. 1I, n = 7) in cells with hTRPA1. Again, this effect lacked a clear concentration dependency (Fig. 1J, n = 5–8). MAA-evoked membrane currents were inhibited by the selective TPRA1-inhibitor HC-030031 (Fig. 1K, n = 6), confirming that the observed effects are generated by hTRPA1. As is demonstrated in Figures 1L and M, both MAA (1 µM) and AA (100 nM) also induced a significant potentiation of carvacrol-induced currents in hTRPA1 cells (MAA: 7 ± 2-fold, n = 7; AA: 5 ± 4-fold, n = 5; *P* < 0.05, paired *t* tests).

**Figure 2.**
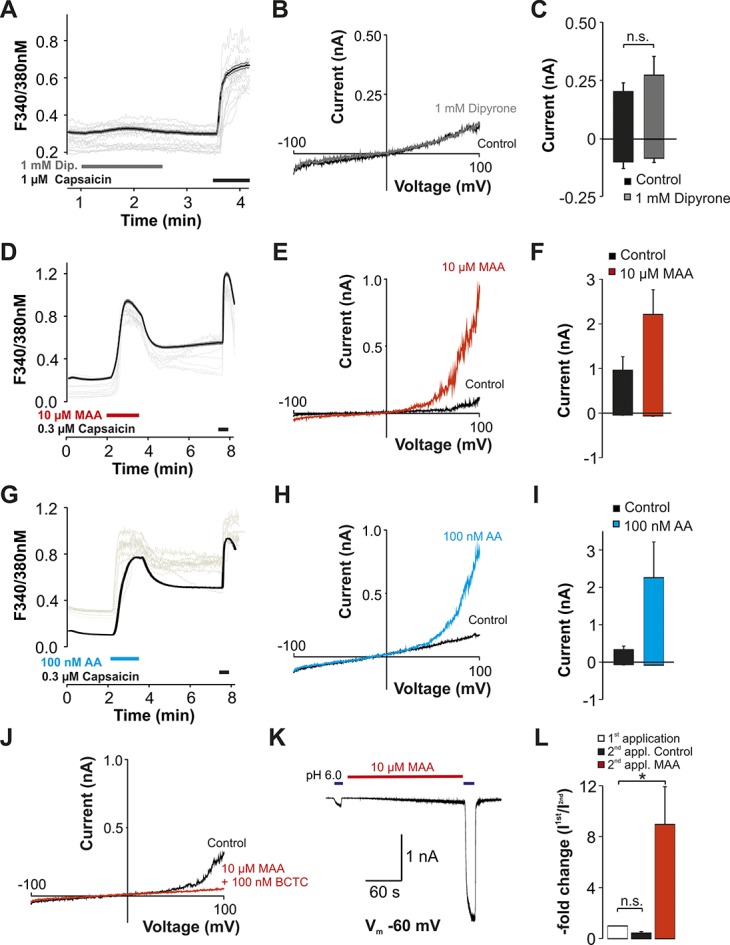
Metabolites of dipyrone activate hTRPV1. (A, D, G) Effects of dipyrone (A, 1 mM), MAA (D, 10 µM), and AA (G, 100 nM AA) on intracellular calcium expressed as ratio F340/380 nm in hTRPV1-HEK 293 cells. Dipyrone and metabolites were applied for 5 minutes followed by application of capsaicin to confirm expression of hTRPV1. The black lines represent the average (mean ± SEM) of recorded cells, and the gray lines represent typical original traces from individual cells. (B, E, H) Representative patch clamp traces during 500 ms-long voltage ramps ranging from −100 mV to 100 mV in HEK293 cells expressing hTRPV1. The ramps were performed during application of either control solution, 1 mM dipyrone (C), 10 µM MAA (F), or 100 nM AA (I). Expression of hTRPV1 was confirmed by application of capsaicin (traces not shown). (C, F, I) Average (mean ± SEM) current amplitudes of currents at −100 and 100 mV during voltage ramps by dipyrone (C, n.s., not significant; Student paired *t* test), MAA (F), and AA (I). (J) Representative patch clamp trace during 500 ms-long voltage ramps ranging from −100 mV to 100 mV in a HEK 293 cell expressing hTRPV1 during application of control solution or MAA in combination with 100 nM of the TRPV1-antagonist BCTC. (K) Representative current traces in hTRPV1-HEK293 cells during 2 consecutive applications of pH 6.0. One micromolar MAA was applied for about 5 minutes between the 2 applications. (L) Average inward currents elicited by the second application of pH 6.0, expressed as average “-fold increase” calculated by normalizing the peak currents amplitudes of the second carvacrol-induced current with the first one. n.s., not significant; Student paired *t* test. *P < 0.05.

Similar to hTRPA1, hTRPV1 was not activated by dipyrone (Fig. 2A, n = 264, Fig. 2B, C, n = 8). However, MAA induced both an increase in intracellular calcium (Fig. 2D, n = 345), and robust membrane currents (Fig. 2E, F, n = 10) in cells expressing hTRPV1. We also observed an activation of hTRPV1 by AA (Fig. 2G, n = 229, Fig. 2H, I, n = 6). The TRPV1-antagonist BCTC (100 nM) inhibited MAA-induced activation (n = 6). MAA also induced a significant potentiation of proton (pH 6.0)-induced currents in hTRPV1 cells (Fig. 2K, L, 9 ± 3-fold, n = 6; *P* < 0.05, paired *t* test).

### 3.2. MAA activates hTRPA1 and hTRPV1 in a redox-dependent manner

**Figure 3.**
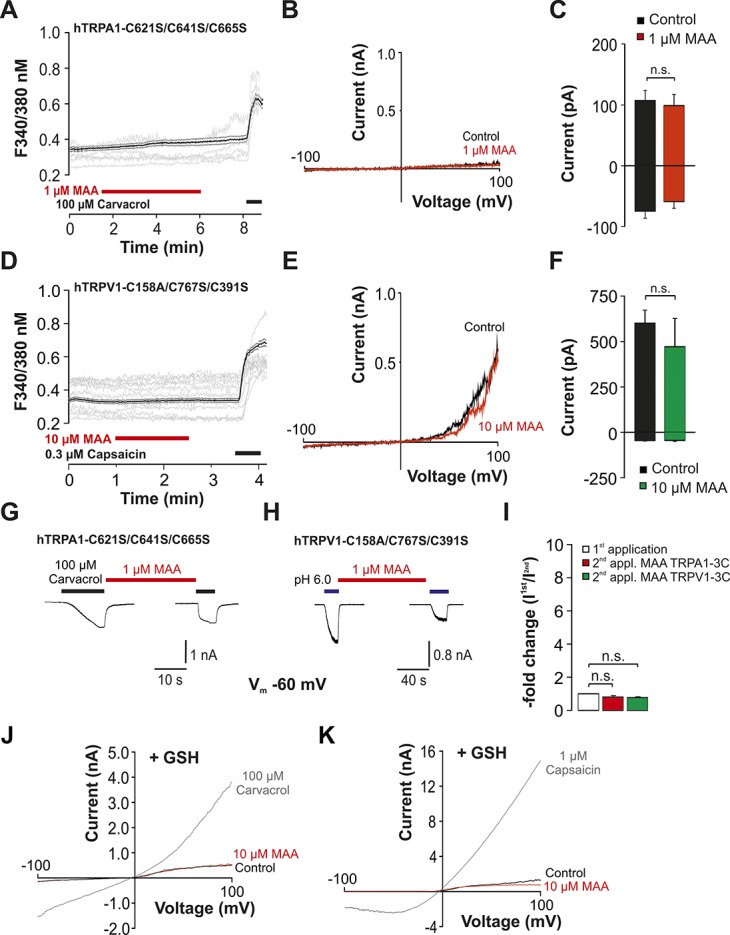
MAA gates hTRPA1 and hTRPV1 through modification of N-terminal cysteines. (A and D) Effects of MAA on intracellular calcium expressed as ratio F340/380 nm in HEK 293 cells expressing hTRPA1-C621S/C641S/C655S (A) or hTRPV1-C158A/C391S/C767S (D). MAA was applied for 5 minutes followed by application of carvacrol respectively capsaicin to confirm expression. The black lines represent the average (mean ± SEM) of recorded cells, and the gray lines represent typical original traces from individual cells. (B and E) Representative patch clamp traces during 500 ms-long voltage ramps ranging from −100 mV to 100 mV in HEK293 cells expressing hTRPA1-C621S/C641S/C655S (B) or hTRPV1-C158A/C391S/C767S (E). The ramps were performed during application of either control solution or MAA. (C and F) Average (mean ± SEM) current amplitudes of currents at −100 and 100 mV during voltage ramps elicited in presence of control solution or MAA. (G and H) Representative current traces of hTRPA1-C621S/C641S/C655S (G) or hTRPV1-C158A/C391S/C767S (H) during 2 consecutive applications of carvacrol and pH 6.0, respectively. MAA was applied for about 5 minutes between the 2 applications. (I) Average inward currents elicited by the second application of carvacrol or pH 6.0, expressed as average “-fold increase” calculated by normalizing the peak currents amplitudes of the second current with the first one. n.s., not significant; Student paired *t* test. (J and K) Representative patch clamp traces during 500 ms-long voltage ramps ranging from −100 mV to 100 mV in cells expressing hTRPA1-C621S/C641S/C655S (J) or hTRPV1-C158A/C391S/C767S (K). The ramps were performed during application of either control solution or 10 µM MAA, and the pipette solution contained 10 mM GSH.

We next asked if MAA might gate TRPA1 and TRPV1 through redox modification. N-terminal cysteines confer both TRPA1 and TRPV1 their redox sensitivity.^2,4,9^ Indeed, MAA failed to evoke a calcium influx (Fig. 3A, n = 77) as well as membrane currents (Fig. 3B, C, n = 7) in cells expressing the redox-insensitive mutant hTRPA1-C621S/C641S/C665S. In case of hTRPV1, the redox-insensitive mutant hTRPV1-C158S/C391S/C767S also failed to generate both MAA-induced calcium influx (Fig. 3D, n = 135) and membrane currents (Fig. 3E, F, n = 6). Furthermore, carvacrol-induced membrane currents were not potentiated by MAA on the mutant hTRPA1-C621S/C641S/C665S (0.8 ± 0.1-fold increase; n = 5, Fig. 3G, I). Accordingly, proton-induced membrane currents were not potentiated by MAA on hTRPV1-C158S/C391S/C767S (75 ± 0.1-fold increase; n = 4, Fig. 3H, I). To substantiate that MAA gates TRPA1 and TRPV1 through oxidation, the antioxidant glutathione (GSH, 10 mM) was added to the pipette solution. Indeed, MAA was completely ineffective on both hTRPV1 and hTRPV1 in presence of intracellular GSH (Fig. 3J, K, n = 5 each).

### 3.3. MAA evokes a calcium influx in mouse dorsal root ganglion neurons

**Figure 4.**
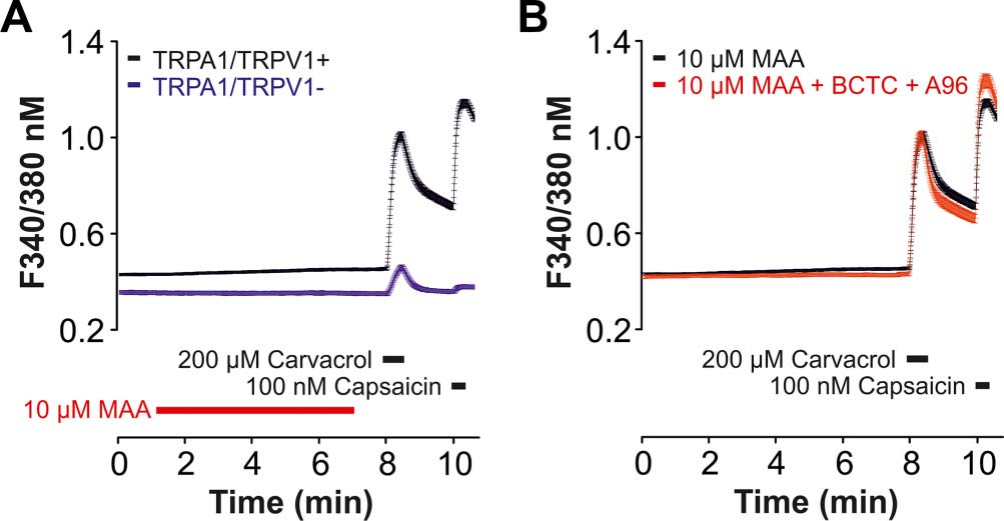
MAA fails to induce a robust calcium influx in mouse DRG neurons. (A and B) Effects of 10 µM MAA on intracellular calcium expressed as ratio F340/380 nm in mouse DRG neurons. MAA was applied for ∼5 minutes followed by application of carvacrol and capsaicin to test expression of TRPA1 and TRPV1. (A) Neurons responding to carvacrol and/or capsaicin (black line) displayed a higher baseline of intracellular calcium and a marginal MAA-induced elevation of calcium as compared to neurons that did not respond to both carvacrol and capsaicin (blue line). (B) Neurons responding to carvacrol and/or capsaicin (black line) displayed a somewhat reduced MAA-induced increase of intracellular calcium in the presence of the TRPA1-inhibitor A-967079 (10 µM) and TRPV1-inhibitor BCTC (100 nM) (red line). The lines represent the average response expressed as mean ± SEM of all recorded cells in each population. DRG, dorsal root ganglion.

We finally asked if TRPA1 and TRPV1 account for a MAA-evoked calcium-influx in mouse DRG neurons. MAA (10 µM) did not evoke a substantial calcium influx in DRG neurons, neither in neurons which responded briskly to carvacrol and/or capsaicin (n = 568), nor in neurons with no or only very small responses to either carvacrol or capsaicin (n = 225). The minimal increase in intracellular calcium occurring throughout the application of MAA in neurons expressing TRPA1 and TRPV1 did not seem to be substantially reduced by the simultaneous inhibition of TRPA1 (A967079, 10 µM) and TRPV1 (BCTC 100 nM) (Fig. 4B, n = 271).

## 4. Discussion

Although associated with severe side effects such as agranulocytosis and with a yet unclear pharmacological mechanism of action, dipyrone is commonly used as a first-choice nonopioid analgesic.^7^ The analgesic effect of dipyrone is strongly reduced in mice lacking TRPA1,^12^ and it was suggested that this effect is due to a dipyrone-induced inhibition of TRPA1. Our data reveal that both MAA and AA gate instead of inhibiting both TRPA1 and TRPV1. This effect seems to be redox dependent and thus involves N-terminal cysteines, which are known to account for gating of both channels by oxidants.^2,4,9^ Although the relevance of these cellular data is yet to be explored, both MAA and AA were already suggested to be required for dipyrone-induced analgesia and antipyresia in rodents.^5,11^ Dipyrone is rapidly hydrolyzed into MAA and then converted into AA after intake, and the protein-unbound plasma levels of MAA and AA at therapeutic dosages of dipyrone are well above the concentrations found to gate TRPA1 and TRPV1.^5,8,15^ Furthermore, the inhibitory effects of MAA and AA on neurons of the rostral ventromedial medulla are reduced by inhibition of TRPV1.^10^ Similar to how acetaminophen was suggested to induce analgesia by inducing a presynaptic inhibition by activating TRPA1 in central nerve terminals,^1^ it seems possible that an activation and sensitization—in addition to an inhibition—of TRPA1 and TRPV1 are relevant for dipyrone-induced analgesia and antipyresia. Further studies are needed to substantiate this somewhat controversial hypothesis.

## Disclosures

The authors have no conflict of interest to declare.
